# Robust Sparse Non-Negative Matrix Factorization for Identifying Signals of Interest in Bearing Fault Detection

**DOI:** 10.3390/s25227041

**Published:** 2025-11-18

**Authors:** Hamid Shiri, Anna Michalak

**Affiliations:** 1 Tony Davies High Voltage Laboratory, School of Electronics and Computer Science, Faculty of Engineering and Physical Sciences, University of Southampton, Southampton SO17 1BJ, UK; h.shiri@soton.ac.uk; 2 Faculty of Geoengineering, Mining and Geology, Wroclaw University of Science and Technology, Na Grobli 15, 50-421 Wrocław, Poland

**Keywords:** bearing fault detection, heavy-tailed noise, non-cyclic impulsive noise, robust sparse non-negative matrix factorization, maximum-correntropy criterion, fault frequency band, Monte Carlo simulation

## Abstract

Bearings are among the most failure-prone components in rotating systems, making early fault detection crucial in industrial applications. While recent publications have focused on this issue, challenges remain, particularly in dealing with heavy-tailed or non-cyclic impulsive noise in recorded signals. Such noise poses significant challenges for classical fault selectors like kurtosis-based methods. Moreover, many deep-learning approaches struggle in these environments, as they often assume Gaussian or stationary noise and rely on large labeled datasets that are rarely available in practice. To address this, we propose a robust sparse non-negative matrix factorization (NMF) method based on the maximum-correntropy criterion, which is known for its robustness in the presence of heavy-tailed noise. This methodology is applied to identify fault frequency bands in the spectrogram of the signal. The effectiveness of the approach is validated using simulated fault signals under both Gaussian and heavy-tailed noise conditions through Monte Carlo simulations. A statistical efficiency analysis confirms robustness to random perturbations. Additionally, three real datasets are used to evaluate the performance of the proposed method. Results from both simulations and real-world data demonstrate the effectiveness of the proposed approach.

## 1. Introduction

Rotating machines play a crucial role in industries, and early fault detection in components such as bearings and gears is essential to prevent catastrophic failures and economic losses. In recent years, numerous studies have been published on this topic, with a particular focus on identifying impulsive behavior in signals caused by faults in rotating systems. However, this impulsive behavior is often obscured by noise, leading many researchers to develop methods for filtering noise to reveal these impulsive components better.

The well-established approach for informative band selection (IFB) is the spectral kurtosis, which offers the advantage of detecting impulsive frequency bands associated with faults while filtering out irrelevant noise [1,2,3,4]. Spectral kurtosis is effective in identifying frequency bands where the signal presents impulsive behavior, which is often associated with faults. However, it has limitations in environments with heavy-tailed impulsive noise or when the artifacts are present in the signal. In those cases, the kurtosis may misinterpret random impulsiveness as fault-related features. In response to these limitations, several alternative approaches have been proposed. Modified sparsity measures such as L-kurtosis, the Gini index, and spectral smoothness have been introduced to improve robustness and allow for detecting the damage also under noisy or repetitive conditions [5,6]. Conditional variance (CV), originally grounded in the statistical interpretation of the 20-60-20 rule, has also proven effective in this context, particularly when implemented in a seven-subset framework tailored for non-Gaussian, impulsive environments [7,8]. Other notable contributions include the use of dependency measures, such as Pearson, Spearman, and Kendall correlations [9], as well as the application of the stability parameter from the α-stable distribution, introduced by Żak et al. [10], both offering alternative paths for identifying frequency bands carrying diagnostically relevant information in complex noise conditions.

Another powerful technique is cyclostationary analysis, which exploits the periodicity inherent in many rotating machine faults [11,12,13,14,15]. This method is particularly useful for detecting cyclic impulsive patterns that occur at specific rotational frequencies. Cyclostationary analysis can enhance fault detection by identifying frequency components modulated by the machine’s operation, but it may struggle with non-cyclic impulsive noise or signals with weak periodicity, limiting its effectiveness in certain noisy environments [16].

In addition to these, signal decomposition methods such as empirical mode decomposition (EMD) [17,18,19], variational mode decomposition (VMD) [20,21,22,23], and local mean decomposition (LMD) [24,25] have gained popularity for fault detection [26,27,28]. These methods decompose a complex signal into simpler components, allowing for the extraction of fault-related features even in the presence of noise. EMD and its variations can adaptively separate non-linear and non-stationary signals into intrinsic mode functions (IMFs), while VMD provides better resolution by isolating specific frequency bands. LMD, on the other hand, is known for its ability to capture local signal variations effectively. Although these methods have proven useful in many scenarios, R. Randall and J. Antoni [29], have demonstrated why the previously mentioned decomposition methods offer limited benefits for bearing diagnostics by highlighting issues such as end effects and mode mixing when detecting bearing faults. In addition, these decomposition methods suffer from heavy-tailed or impulsive noise.

Deep-learning approaches have achieved notable success in fault diagnosis. Architectures tailored to rotating machinery, such as MHSNet, also reach high accuracy through multi-sensor fusion and long-range dependency modeling [30], while operator-learning frameworks like the Generalized Koopman Neural Operator demonstrate efficient data-driven modeling of railway pantograph–catenary dynamics [31]. To address data scarcity, Yan et al. [32] proposed a multimodal arc-detection network based on diffusion models that is explicitly designed for limited-data railway systems, leveraging unlabeled data and audio–visual fusion to improve robustness. Nevertheless, despite these advances, deploying supervised or semi-supervised deep models in our setting still requires modality-specific training and extensive label curation. More importantly, most deep-learning architectures rely on statistical assumptions—such as approximately Gaussian, stationary, and independently distributed noise—that are often violated by heavy-tailed and non-cyclic impulsive noise commonly observed in vibration and acoustic measurements. In many industrial systems, such as crushers or belt conveyors, these impulsive or outlier events are inherent to the mechanical process itself rather than removable anomalies, making conventional outlier suppression inappropriate and potentially destructive to informative features. Such conditions lead to unstable feature extraction, biased gradient updates, and poor generalization in deep-learning models.

Non-negative matrix factorization (NMF) and non-negative tensor factorization (NTF) have been widely applied to fault detection, particularly using spectrogram representations as inputs [33,34,35,36,37]. The spectrogram, which provides a time-frequency representation of the signal, is highly advantageous in fault detection because it captures both the frequency and temporal characteristics of faults, making it ideal for analyzing rotating machinery signals. By using the spectrogram as input, NMF and NTF can decompose the signal into distinct frequency bands and time components, offering a clearer understanding of the underlying fault-related structures. M. Gabor et al. [38] developed stochastic sampled orthogonal non-negative matrix factorization (SS-ONMF) to extract frequency-based features from the spectrogram of measured signals. Their proposed algorithm identifies a selective filter specifically tailored to the frequency band of the SOI. The results presented emphasize the importance of orthogonality constraints in producing highly selective filters that effectively capture only the informative frequency band. A. Michalak et al. [39] investigated the impact of heavy-tailed noise on the extraction of the SOI using NMF. They conducted an empirical analysis to evaluate how different noise levels affect the effectiveness of SOI extraction with two NMF algorithms: a multiplicative algorithm that minimizes the Euclidean objective (Standard NMF) and a generalized version of the hierarchical alternating least squares algorithm (β-HALS) for minimizing Beta-divergence. Their findings from simulations and real data analysis demonstrated that the β-HALS NMF algorithm outperforms standard NMF in terms of extraction efficiency.

However, all the mentioned algorithms, which are typically based on least-squares cost functions or divergence-based approaches, are sensitive to heavy-tailed noise and outliers, which can distort the decomposition results and lead to inaccurate fault detection.

To overcome this limitation, we propose a procedure using the robust sparse NMF method, based on the MCC, which offers enhanced robustness against outliers and heavy-tailed noise compared to traditional least-squares-based approaches. MCC leverages similarity measures rooted in higher-order statistics, making it more effective in handling heavy-tailed noise, as it downplays large deviations that are typical in such noise distributions. Additionally, the inclusion of a sparsity constraint focuses the decomposition on the most relevant components, allowing for a more precise extraction of key features. This is particularly advantageous for detecting impulsive signals, as the sparsity encourages the model to isolate sharp, localized events, thereby improving the identification of meaningful patterns hidden in noisy data. Additionally, NNDSVD [40] initialization is used in our proposed method, providing an accurate starting point compared to random initialization, which leads to faster convergence and improved decomposition accuracy. This ensures that the algorithm captures key components more reliably, further enhancing its ability to detect impulsive signals, especially in noisy environments. Additionally, the procedure utilized the Envelope Spectrum Indicator (ENVSI) to identify which filters from the NMF family effectively capture the fault. In summary, the contributions of this work can be summarized as follows:

1. **Robust cost function:** In the literature, various NMF frameworks and robust cost functions have been explored for bearing condition monitoring. In this work, we further adapt the NMF framework to this purpose by introducing the **Maximum Correntropy Criterion (MCC)**, which significantly improves robustness against heavy-tailed and impulsive noise compared with classical least-squares- and β-divergence-based NMF methods.
2. **Sparsity constraint:** An $\ell_{1}$-norm sparsity term is incorporated into the NMF formulation to emphasize localized impulsive components that characterize bearing defects, enhancing the clarity and separability of cyclic features.
3. **Stable initialization:** The **NNDSVD initialization** is applied to both W and H matrices to ensure stable and reproducible decompositions and to accelerate convergence of the algorithm.
4. **Automatic filter selection:** The **Envelope Spectrum Indicator (ENVSI)** is employed to automatically identify the most informative NMF component corresponding to the fault-related frequency band, ensuring objective and repeatable filter selection.
5. **Comparative validation:** The proposed **Sparse NMF-MCC** is benchmarked against the **classic NMF** and **β-HALS NMF** algorithms, demonstrating that the MCC-based approach consistently achieves higher ENVSI values and clearer fault-frequency localization across both simulated and real-world datasets (belt conveyor, ore crusher, and test rig).

The paper is organized as follows: After the introduction, Section 2 presents the critical components of the proposed approach in theory. Then, in Section 3, the suggested model is simulated, and the results are presented. The results of applying the proposed approach to three benchmark data sets are presented in Section 4, along with an indication of all intermediate steps. Finally, the conclusions are formed in Section 5.

## 2. Methodology and Theory

**Figure 1:**
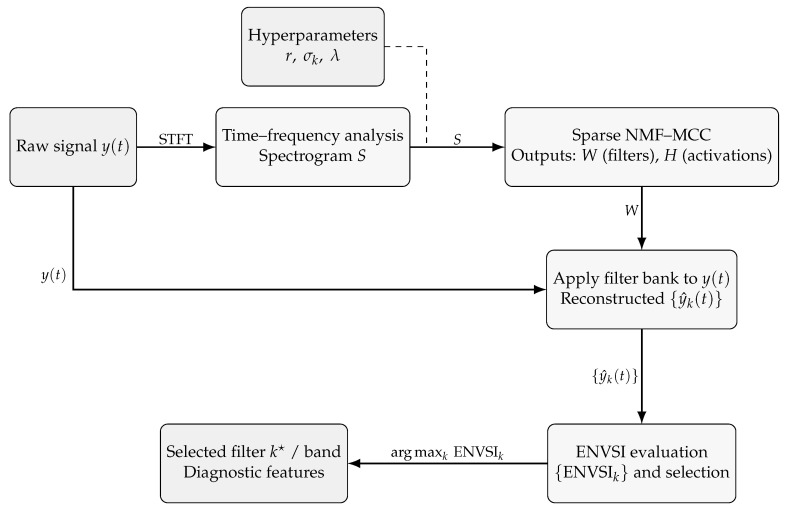
Framework of the proposed Sparse NMF–MCC pipeline. Inputs: raw signal y(t) and hyperparameters $\left(r,\sigma_{k},\lambda \right)$. The spectrogram *S* is factorized into basis *W* and activations *H*. Filters derived from *W* are applied to y(t) to obtain reconstructed signals $\left\{{\hat{y}}_{k}\left(t\right)\right\}$, which are scored by ENVSI; the component with the highest ${\mathrm{ENVSI}}_{k}$ is selected for diagnostics.

The proposed methodology’s framework is illustrated in Figure 1. The process starts with the input of the raw signal, followed by the configuration of key parameters: the number of filters or rank of decomposition (*r*), the maximum correntropy bandwidth ($\sigma_{k}$), the sparsity coefficient (λ), and the number of iterations. Next, a spectrogram of the raw signal is computed and decomposed using the proposed Sparse NMF-MCC method, resulting in the basis matrix W and the coefficient matrix H.

Each basis vector in W is then used to filter the raw signal, producing a set of candidate filtered signals. To automate the selection of the most informative filter, the ENVSI is computed for each filtered signal. The filtered signal with the highest ENVSI value is selected as the final output, as it most clearly emphasizes the fault-related spectral components. Each step of the proposed methodology is described in detail in the following subsections.

A complete list of symbols and parameters used in the formulas is provided in Appendix B (Table A1).

### 2.1. Short-Time Fourier Transform (STFT)

The short-time Fourier transform (STFT) of a one-dimensional discrete data **y** is defined as [41]:

(1) $${\mathrm{STFT}}_{t,f}=\sum_{n=0}^{L-1}y\left[n\right]w\left[n-t\right]e^{-\frac{j2\pi fn}{N}},$$

where w[n−t] is a window function centered around *t* used to localize the signal in time, L is the number of samples in the analyzed segment of the signal (window length), N is the number of points used for the Discrete Fourier Transform, *f* is the frequency, and *j* is the imaginary unit, with $j^{2}=-1$.

The spectrogram **S** is the absolute value of **STFT**, forming a non-negative matrix that represents the time-frequency distribution of a signal. The window function w[n−t] determines the resolution trade-off between time and frequency: a narrow window yields better time resolution but poorer frequency resolution, while a wide window offers better frequency resolution but poorer time resolution.

### 2.2. NMF Model

Non-negative Matrix Factorization (NMF) is a technique used to decompose a non-negative data matrix S into a product of two lower-rank non-negative matrices: W (the basis matrix) and H (the coefficient matrix). In general, the NMF model can be expressed as follows [42,43,44,45]:

(2) S=WH+E,

where $S\in R_{+}^{m\times n}$ is the input matrix, $W\in R_{+}^{m\times r}$ (the basis matrix) and $H\in R_{+}^{r\times n}$ (the coefficient matrix), $E\in R^{m\times n}$ is the error matrix and r is the rank of the factorization. In the proposed approach, the input matrix with non-negative entries is the spectrogram matrix. One of the resulting matrices, the base matrix **W**, can be used as a filter characteristic. This allows the filtering of the signal and enables the identification of the faults.

### 2.3. Maximum Correntropy Criterion (MCC) with Gaussian Kernel

Let $x=\{x_{1},x_{2},\ldots ,x_{N}\}$ and $y=\{y_{1},y_{2},\ldots ,y_{N}\}$ represent two sets of random variables. The correntropy between x and y is defined as [46,47]:

(3) $$V\left(x,y\right)=E\left[\kappa_{\sigma_{k}}\left(x-y\right)\right],$$

where E[·] denotes the expectation operator and $\kappa_{\sigma_{k}}\left(\cdot \right)$ is a Gaussian kernel function given by:

(4) $$\kappa_{\sigma_{k}}\left(x-y\right)=\exp\left(-\frac{{\left(x-y\right)}^{2}}{2\sigma_{k}^{2}}\right),$$

with $\sigma_{k}>0$ as the kernel bandwidth parameter.

The Gaussian kernel in MCC has two important properties that make it an effective tool in analyzing data with noise:

- **Robustness**: The Gaussian kernel suppresses large deviations by assigning lower values to them. For large deviations, the value of $\exp\left(-\frac{{\left(x-y\right)}^{2}}{2\sigma_{k}^{2}}\right)$ approaches zero, making corentropy less sensitive to outlier points than traditional measures such as mean squared error, which enhances the robustness of MCC against outliers.
- **Bandwidth Control**: The parameter $\sigma_{k}$ dictates the width of the Gaussian kernel, balancing between sensitivity to small errors and robustness against large ones.

In the classical NMF method, the data matrix **S** is approximated by the product of two non-negative matrices **W** and **H**, minimizing the distance between **S** and **WH**, usually using Euclidean, Kullback-Leibler, or Itakura-Saito divergence. In optimization problems, MCC maximizes the correntropy between the original signal and the output one. In the MCC-NMF approach, the cost function is replaced by a measure of correntropy [48]. It can be written as follows:

(5) $$\max_{W,H}V\left(S,\mathrm{WH}\right)=E\left[\exp\left(-\frac{{\left(S-\mathrm{WH}\right)}^{2}}{2\sigma_{k}^{2}}\right)\right].$$

### 2.4. Sparse NMF with MCC Objective

Sparse NMF is an extension of the NMF method that introduces additional sparsity constraints during matrix decomposition [49]. The proposed approach uses the most popular method for introducing sparsity in NMF, i.e., adding a penalty term in the $l_{1}$ norm to the cost function.

(6) $$\max_{W,H\geq 0}\sum_{i=1}^{m}\sum_{j=1}^{n}\exp\left(\frac{{\left(S_{ij}-{\left[\mathrm{WH}\right]}_{ij}\right)}^{2}}{2\sigma_{k}^{2}}\right)-\lambda_{H}{∥H∥}_{1},$$

where $\sigma_{k}>0$ represents the bandwidth of the Gaussian kernel used in the MCC, $\left|\right|\cdot {\left|\right|}_{1}$ means the $l_{1}$ norm, $\lambda_{H}$ is the regularization parameter controlling the sparsity of H. The term $\exp\left(\frac{{\left(S_{ij}-{\left[\mathrm{WH}\right]}_{ij}\right)}^{2}}{2\sigma_{k}^{2}}\right)$ represents the Gaussian kernel applied to the residuals, while the regularization term $\lambda_{H}{∥H∥}_{1}$ promotes sparsity in H, encouraging solutions with fewer active components.

### 2.5. Algorithm of Sparse NMF MCC

The MCC weight Ω is calculated based on the residual $E_{ij}=S_{ij}-{\left(\mathrm{WH}\right)}_{ij}$ and is given by:

(7) $$\Omega_{ij}=\exp\left(-\frac{{\left(S_{ij}-{\left[\mathrm{WH}\right]}_{ij}\right)}^{2}}{2\sigma_{k}^{2}}\right)=\exp\left(-\frac{E^{2}}{2\sigma_{k}^{2}}\right).$$

This weight Ω is used to modify the update rules of **W** and **H**. They help adjust the importance of individual approximation errors, down-weight significant reconstruction errors, and provide robustness to outliers.

The algorithm implements Sparse NMF MCC using multiplicative update rules for W and H. It consists of three main stages, namely initialization, update rules, and stopping condition.

- **Initialization:** Initialize the matrices W and H with the non-negative values. Set the parameters $\sigma_{k}$ and λ. In presented case both matrices, i.e., W and H were initialize using NNDSVD.
- **Update Rules:** The update for H incorporates the MCC weights:
  (8) $$H\leftarrow H⊙\frac{W^{T}\left(\Omega ⊙\left(S⊘\left(\mathrm{WH}+\epsilon \right)\right)\right)}{1+\lambda },$$
  where $W^{T}$ is the transposition of matrix W, Ω is the matrix of MCC weights, ⊙ is the Hadamard product, ⊘ is the Hadamard division and ϵ is a small constant to prevent division by zero. The update for W is:
  (9) $$W\leftarrow \frac{W⊙\left(\Omega ⊙\left(S⊘\left(\mathrm{WH}+\epsilon \right)\right)\right)H^{T}}{\left|\right|H^{T}{\left|\right|}_{1}}$$
  Finally, the normalization of **W** is done by the columns of matrix **W**:
  $$W\leftarrow \frac{W}{{\left||W|\right|}_{1}}.$$
  In this paper, two versions of the Sparse NMF MCC are discussed. In the first version, it is assumed that the kernel size (bandwidth) is a constant parameter. In the second version, the kernel size is calculated using a following formula [48,50]:
  (10) $$\sigma_{k}^{2}=\frac{1}{mn}\sum_{i=1}^{m}\sum_{j=1}^{n}{\left(S_{ij}-{\left[\mathrm{WH}\right]}_{ij}\right)}^{2}$$
- **Stopping Condition:** The algorithm continues until a maximum number of iterations is reached or the optimality gap becomes sufficiently small.

Additionally, Algorithm 1 provides the pseudocode detailing the proposed methodology for reference.

The described Sparse NMF MCC is particularly effective in environments with heavy-tailed noise or outliers, which are commonly found in real-world applications, such as vibration signal analysis. By applying the Sparse NMF with MCC to the spectrogram matrix **S** of a vibration signal, the algorithm can isolate specific frequency components related to cyclic impulses while simultaneously suppressing non-cyclic noise and other irrelevant signal components. The sparsity constraint in **H** helps focus on the impulsive nature of the signal, making this method ideal for detecting cyclic impulsive signals in noisy environments.

For clarity and consistency, the list of all variables and parameters appearing in the formulas is provided in Appendix B.

### 2.6. Selecting the Parameters

Another important aspect of the proposed model is the selection of hyperparameters, such as the MCC bandwidth ($\sigma_{k}$), sparsity constraint (λ), and the number of filters (NMF rank *r*), all of which significantly influence the final results. The bandwidth of MCC ($\sigma_{k}$) plays a crucial role in balancing sensitivity and robustness—smaller $\sigma_{k}$ values increase robustness by reducing the influence of outliers and heavy-tailed noise, while larger $\sigma_{k}$ values make the method more sensitive to noise. The sparsity constraint (λ) dictates the emphasis placed on sparsity; a higher λ results in a more focused decomposition on key components, which is beneficial for detecting impulsive signals. However, excessive sparsity may overlook important details. The rank of NMF (*r*) determines the number of filters or components; a higher *r* provides more detail but may lead to overfitting, while a lower *r* simplifies the decomposition but risks missing nuances.

Balancing these hyperparameters is key to optimizing the performance of Sparse NMF MCC. The bandwidth $\sigma_{k}$ controls noise management, λ regulates the sparsity level, and *r* defines the resolution of the decomposition—each influencing the quality of cyclic impulsive signal detection. In this work, these parameters were selected through trial and error, following the described rules. Still, there is considerable potential to apply constrained optimization algorithms for choosing the optimal values. The ENVSI (defined in Section 2.7) could serve as the output metric to be maximized, with $\sigma_{k}$, λ, and *r* as input parameters to be optimized for the best performance. It is also worth mentioning that the effectiveness of MCC bandwidth $\sigma_{k}$ has been investigated in several previous studies [51,52].
**Algorithm 1:** Sparse NMF MCC: Sparse Non-negative Matrix Factorization with Maximum Correntropy Criterion
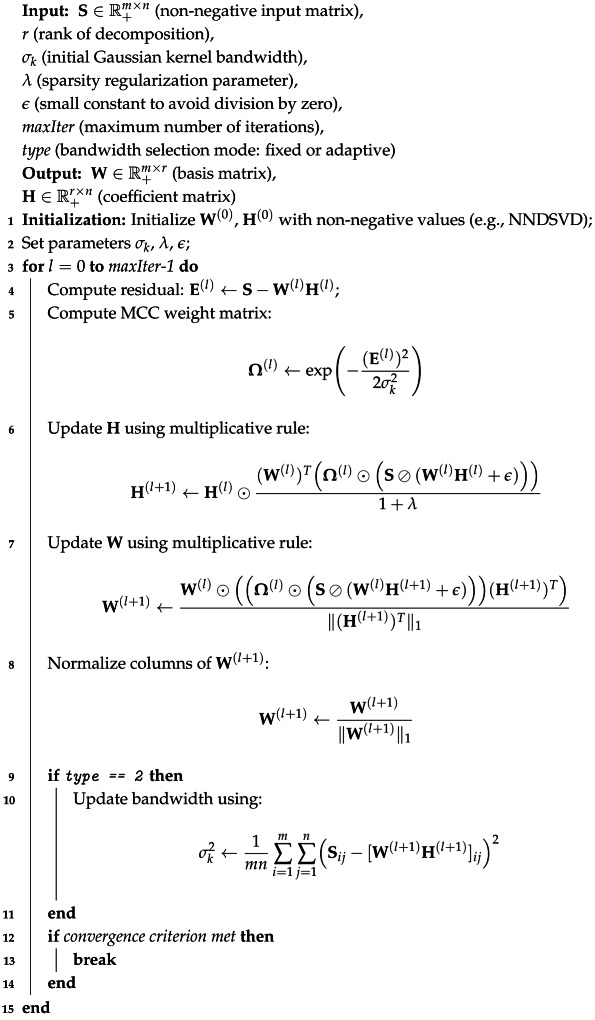


### 2.7. Envelope Spectrum Indicator

The Envelope Spectrum Indicator, called ENVSI, is suitable due to its ability to emphasize fault-related spectral components in a clear and quantifiable manner. It compares the energy of the components corresponding to the fault to the energy of the whole signal at a given frequency range. In the proposed work, it has been utilized in two ways: to select the NMF class associated with the damage signature, and to compare the performance of the proposed methods with that of the benchmark techniques. It takes values between 0 and 1, resulting in simplicity of interpretation. A higher ENVSI value indicates a clearer representation of the fault frequency and its harmonics, confirming the relevance of the extracted features. In contrast, a low ENVSI value means that the method does not effectively isolate the informative frequency band, i.e., the damage cannot be detected. The ENVSI can be defined as follows [8]:

(11) $$\mathrm{ENVSI}=\frac{\sum_{i=1}^{H}A_{\mathrm{fault},i}^{2}}{\sum_{j=1}^{N}{\mathrm{SES}}_{j}},$$

where $A_{\mathrm{fault},i}$ denotes the amplitude of the envelope spectrum at the fault frequency and its corresponding harmonics, while *H* refers to the number of harmonics included in the analysis. In the presented analysis, the H is equal to 5. The denominator, *N*, represents the total number of frequency bins used to compute the overall energy in the squared envelope spectrum (SES).

## 3. Simulation

This section outlines the procedure for generating synthetic signals under various conditions, including Gaussian noise with different SNR levels and heavy-tailed noise with varying intensities. The proposed method is then applied to these signals to evaluate its robustness and effectiveness under diverse noise environments.

To benchmark the performance of the proposed approach, several established NMF techniques were employed for comparison. In particular, the Classic NMF method was implemented using the nnmf function available in MATLAB 2024a [53]. Additionally, the β-HALS NMF algorithm, as presented in [39], was considered.

### 3.1. Simulated Signal

In the following, the equations for the generated synthetic signal are presented. The observed signal, y, in a vibration-based fault detection scenario, can be modeled as the superposition of two components:

(12) y=s+n,

where **y** represents the total simulated signal, s denotes the SOI component, and n describes the noise. The noise n is classified into two categories: zero-mean white Gaussian noise and heavy-tailed noise. For the heavy-tailed distribution, the Student’s t-distribution has been selected.

The Student’s t distribution is a fundamental statistical tool to incorporate an essential parameter: degrees of freedom ν. This distribution provides a robust framework for modeling data, accommodating deviations from normality. Widely utilized in statistical inference, hypothesis testing, and modeling scenarios involving small sample sizes or unknown population standard deviation, Student’s t distribution enhances the accuracy and reliability of statistical conclusions. The probability density function of the Student’s t distribution is expressed as follows:

(13) $$f\left(x;\nu \right)=\frac{\Gamma \left(\frac{\nu +1}{2}\right)}{\sqrt{\nu \pi }\;\Gamma \left(\frac{\nu }{2}\right)}{\left(1+\frac{x^{2}}{\nu }\right)}^{-\frac{\nu +1}{2}}.$$

**Figure 2:**
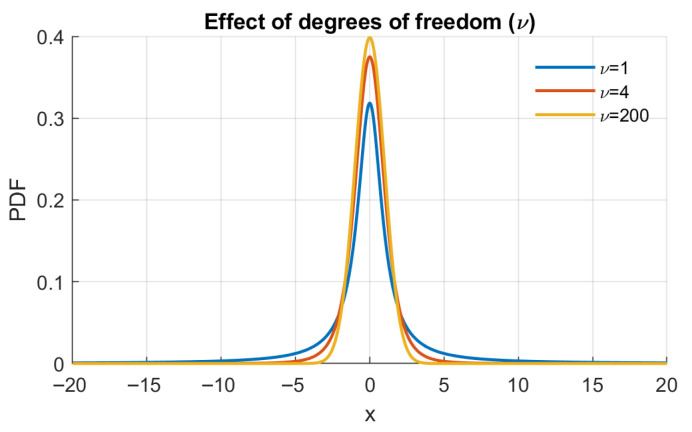
The effect of degree of freedom ν on Student t distribution.

In this expression, *x* represents the random variable, and ν denotes the degrees of freedom. For a visual representation of the impact of the degrees of freedom parameter on the probability density function of the Student’s t distribution, refer to Figure 2.

The SOI, s, is characterized as a periodic impulse signal. It is modeled using a damped sinusoidal function that repeats periodically over time. The mathematical representation of s is given as:

(14) $$s\left(t\right)=A\sum_{m=-\infty }^{\infty }\exp\left\{-\alpha_{s}t\right\}\sin\left(2\pi f_{c}t+\phi_{s}\right)*\delta \left(t-m\left(T_{p}+\Delta T_{p}\left(t\right)\right)\right).$$

In this formulation, *A* denotes the magnitude of the sinusoidal component, while $f_{c}$ specifies the frequency. The damping factor $\alpha_{s}$ indicates the rate at which the amplitude decays over time. The function δ(·) is the Dirac delta function, which localizes the impulses. The term $\phi_{s}$ represents the initial phase shift. The convolution operation is indicated by the symbol *. The nominal period of the signal is denoted as $T_{p}$, and $\Delta T_{p}\left(t\right)$ describes the slow variations in the period due to jitter effects.

### 3.2. Signal in the Presence of Gaussian Noise

**Figure 3:**
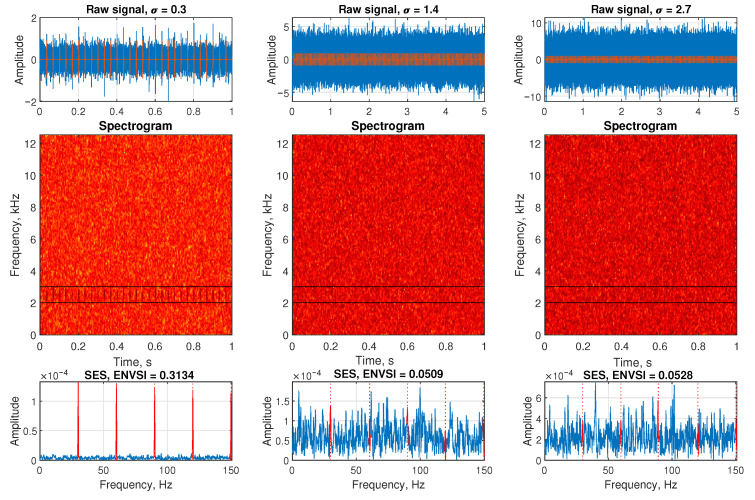
Simulated signals in the presence of Gaussian noise, spectrograms, and SES for three different σ parameters, respectively.

Figure 3 illustrates a synthetic signal contaminated with Gaussian noise at three different levels. The SOI is centered at 2500 Hz and includes a periodic impulsive component.

The top row presents the raw signals. In the first subplot, the noise level is relatively low (σ=0.3), allowing the impulsive component to be partially visible despite a modest SNR. In the middle and right subplots, the noise levels increase to σ=1.4 and σ=2.7, respectively, resulting in increasingly challenging SNRs where the impulsive features become difficult to distinguish.

The middle row shows the corresponding spectrograms, computed using MATLAB’s spectrogram function with parameters detailed in Table 1. For the low-noise case (σ=0.3), the impulsive components around the 2500 Hz center frequency are clearly isolated in the time-frequency domain. However, as the noise level increases, the spectrograms become dominated by noise, and the periodic components are no longer discernible—highlighting the limitations of conventional spectral methods under low SNR conditions.

The bottom row presents the squared envelope spectrum (SES) for each case. For the low-noise case, clear periodic peaks appear in the SES, and the ENVSI value is relatively high, indicating strong spectral sparsity and the presence of modulation. Conversely, for σ=1.4 and σ=2.7, the SES becomes flatter and less structured, resulting in much lower ENVSI values. This underscores the need for more advanced signal processing methods to enhance and extract impulsive features under high-noise conditions.

**Figure 4:**
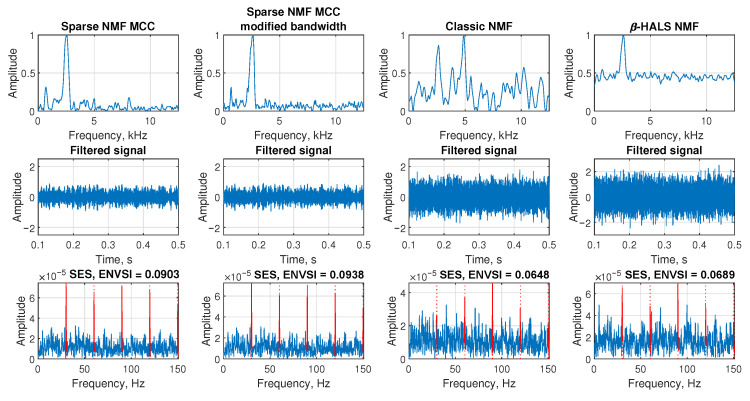
The comparison of the results obtained from the Sparse NMF MCC, Sparse NMF MCC with the modified bandwidth, classic NMF, and β-HALS NMF, respectively. The analysis is presented from the exemplary signal with Gaussian noise (σ = 1.4, SNR = −21.678).

The Figure 4 is presented based on an exemplary signal contaminated by Gaussian noise with standard deviation σ=1.4, corresponding to a low signal-to-noise ratio of SNR = −21.678 dB. The aim is to compare the performance of different NMF-based filtering techniques in extracting the impulsive component from this noisy signal.

In the first row, the the filter spectral profiles of the extracted components are shown. The first subplot illustrates the frequency response of the Sparse NMF MCC method, where the band-pass component around the frequency of interest (2500 Hz) is clearly visible. The second subplot shows the result of the Sparse NMF MCC with modified bandwidth, which still preserves good selectivity around the main spectral region. In contrast, the third subplot (Classic NMF) shows multiple wideband and less structured components, and in the last subplot the β-HALS NMF method detects the correct bandwidth; however, the amplitude of the baseline is relatively high, indicating that this type of filter tends to retain a significant amount of background noise.

In the second row, the corresponding filtered signals are presented in the time domain. The third row shows the SES and the corresponding ENVSI value calculated for each method. For the Sparse NMF MCC method and Sparse NMF MCC with modified bandwidth, the periodic peaks in SES are clearly visible, and ENVSI reaches values of 0.09303 and 0.0938, respectively. On the other hand, Classic NMF and β-HALS NMF methods fail to highlight the periodic structure, resulting in lower ENVSI values of 0.0648 and 0.0689. These results demonstrate that under low SNR conditions, Sparse NMF MCC-based methods provide superior filtering capability and improved impulsive feature extraction.

**Figure 5:**
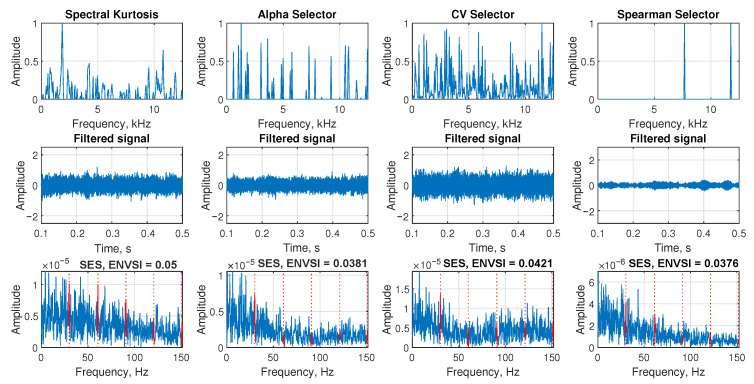
The comparison of the results obtained from the spectral kurtosis, Alpha selector, CV selector, and Spearman selector, respectively. The analysis is presented from the exemplary signal with Gaussian noise (σ = 1.4, SNR = −21.678).

The results shown in Figure 5 were calculated using the same signal as in Figure 4. This allows for a comparison of the outcomes obtained by the NMF-based approaches. The results are presented in the same scheme.

In the first row, the spectral kurtosis, Alpha selector, CV selector, and Spearman selector are presented, respectively. Unfortunately, none of these selectors provides the correct informative frequency band, making it impossible to determine whether the signal contains damage. The second row presents the corresponding filtered signals in the time domain, which also do not convey any information about the damage. The third row illustrates the SES and the corresponding ENVSI values calculated for each method. The peaks corresponding to the fault frequency and their harmonics are not visible, and the ENVSI values are low.

### 3.3. Signal in the Presence of Heavy-Tailed Noise

**Figure 6:**
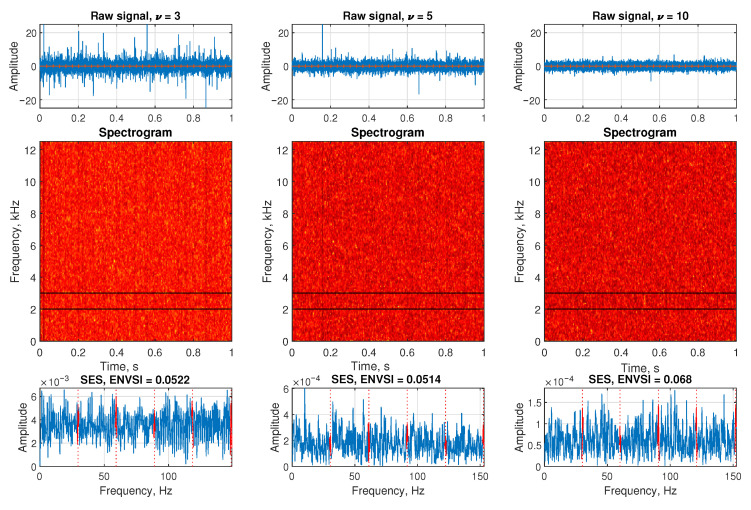
Simulated signals in the presence of heavy-tailed noise (Student’s t with three different ν parameter), spectrograms and SES.

The Figure 6 is presented to investigate the effect of heavy-tailed noise on the signal characteristics. In this case, the raw signal is contaminated with impulsive noise modeled using a Student’s *t*-distribution with varying degrees of freedom ν∈{3,5,10}. The parameter ν controls the tail behavior of the distribution: lower values correspond to heavier tails and more extreme noise outliers, while higher values approach Gaussian behavior.

In the first row, the raw signals are shown. As we can see, when ν=3, the signal is heavily corrupted by high-amplitude fluctuations due to the impulsive nature of the noise. As ν increases to 5 and then to 10, the impulsiveness of the noise decreases, and the amplitude range becomes more consistent with Gaussian-like behavior. However, in all three cases, the impulsive components of interest are difficult to observe directly from the time domain due to the presence of strong noise.

The second row presents the spectrograms calculated for each signal using MATLAB’s spectrogram function with the same parameters described in Table 1. In all three subplots, the time-frequency representation is dominated by broadband noise, and there is no clear evidence of the periodic impulsive structure. This highlights the challenge of detecting fault-related features under heavy-tailed, non-Gaussian noise conditions.

In the third row, the SES is calculated for each case, and the corresponding ENVSI values are also shown. As expected, for ν=3, the SES is highly irregular and noisy, with no significant peaks, and the ENVSI value is low (0.0522). Similarly, for ν=5 and ν=10, the ENVSI values remain low (0.0514 and 0.068, respectively), and the periodic structure is not captured. These results confirm that in the presence of heavy-tailed impulsive noise, traditional energy-based spectral features such as SES and ENVSI are ineffective for detecting weak, periodic fault signatures, motivating the need for more robust and adaptive signal processing techniques.

**Figure 7:**
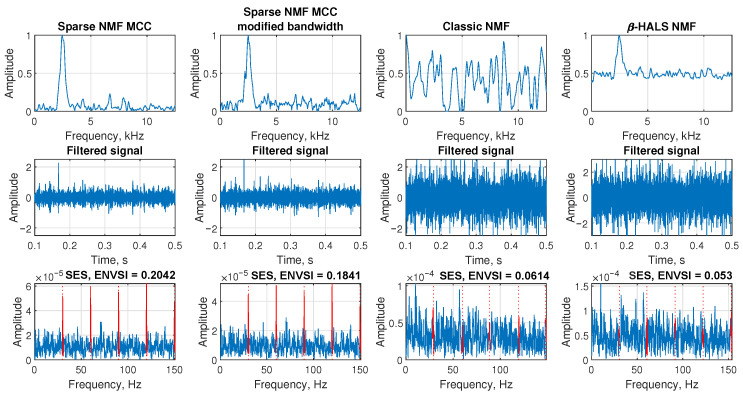
The comparison of the results obtained from the Sparse NMF MCC, Sparse NMF MCC with the modified bandwidth, classic NMF, and β-HALS NMF, respectively. The analysis are presented from the exemplary signal with heavy-tailed noise (ν=5).

The Figure 7 presents the performance comparison of different NMF-based filtering methods when applied to a signal contaminated with heavy-tailed noise modeled by a Student’s *t*-distribution with degrees of freedom ν=5. This case represents a strong impulsive noise environment that challenges traditional filtering approaches.

In the first row, the filter spectral profiles of the extracted components are shown. For the Sparse NMF MCC method, the frequency band around the target component is well identified. Similarly, in the second subplot, the modified bandwidth version of Sparse NMF MCC also captures the relevant band, although it exhibits a broader shape. The Classic NMF method (third subplot) produces the chaotic filter spectral profile. In the last subplot, the β-HALS NMF detects a narrow region of interest, but with a high baseline amplitude, which suggests that it retains a considerable amount of background noise.

The second row shows the time-domain filtered signals. In both Sparse NMF MCC methods, impulsive features are partially preserved despite the high noise level, and the background noise is relatively reduced. However, Classic NMF and β-HALS NMF fail to suppress the heavy-tailed noise sufficiently, making it difficult to distinguish impulsive components from the output signal.

In the third row, the SES and the ENVSI values are calculated. The highest ENVSI is obtained using Sparse NMF MCC (0.2042), followed closely by its modified bandwidth version (0.1841), both showing distinguishable periodic peaks. On the contrary, Classic NMF and β-HALS NMF result in noisy SES plots with weak or invisible periodic structure, and their ENVSI values drop to 0.0614 and 0.053, respectively. These findings suggest that under the influence of heavy-tailed noise, Sparse NMF MCC methods are more robust in isolating modulated fault-related components.

**Figure 8:**
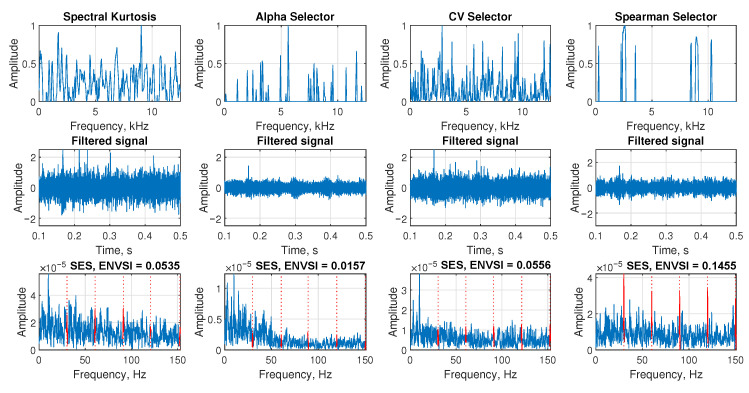
The comparison of the results obtained from the spectral kurtosis, Alpha selector, CV selector, and Spearman selector, respectively. The analysis is presented from the exemplary signal with heavy-tailed noise (ν=5).

The results presented in Figure 8 were obtained using the same signal as in Figure 7. This enables a comparison of the outcomes achieved by the NMF-based approaches. In the first three subplots, which correspond to spectral kurtosis, the Alpha selector, and the CV selector, the filters obtained appear random and do not indicate any damage. However, the Spearman selector shows a partially correct band. The second row displays the time-domain filtered signals, while the third row presents the SES and ENVSI values. In the first three subplots, where the filters do not provide the correct IFB, the SES are noisy, and the periodic component cannot be visible. The ENVSI values are low. In contrast, the SES corresponds to the Spearman selector has a high noise level; however, the periodic impulses corresponding to the fault frequency and its harmonics, marked in red, are visible. This results in the ENVSI value (0.1455) being the highest among all the compared selectors. However, it is still lower than the one obtained based on the Sparse NMF MCC and its modified bandwidth version.

### 3.4. Monte-Carlo Analysis for Simulated Signal

In this section, a Monte Carlo analysis is carried out to evaluate the robustness and repeatability of the proposed filtering approaches under various noise conditions. By performing multiple independent simulations with different realizations of noise, both Gaussian and heavy-tailed (Student’s *t*-distributed), we aim to assess the consistency of frequency localization and fault feature extraction across different NMF-based methods.

### 3.5. Gaussian Case

**Figure 9:**
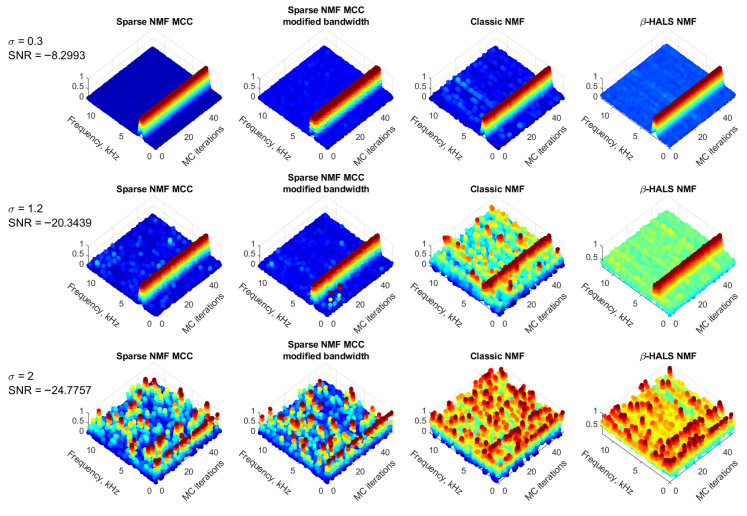
50 Simulated signals in the presence of a different range of Gaussian noise.

The Figure 9 presents a Monte Carlo analysis conducted over 50 independent trials to assess the robustness and consistency of the proposed filtering methods under different levels of Gaussian noise. Three different values of the noise standard deviation are considered: σ=0.3, σ=1.2, and σ=2.0, corresponding to SNR levels of −8.2993 dB, −20.3439 dB, and −24.7757 dB, respectively. The objective is to evaluate how each method behaves across repeated runs and whether it can consistently extract the impulsive component under varying noise conditions.

### 3.6. Non-Gaussian Case

Each row corresponds to a different noise level. The columns compare the four filtering approaches: Sparse NMF MCC, Sparse NMF MCC with modified bandwidth, Classic NMF, and β-HALS NMF. The vertical axis shows the normalized amplitude of the extracted components, plotted across frequency (horizontal axis) and Monte Carlo iterations (depth axis).

In the top row (σ=0.3, high SNR), all methods can localize the frequency component of interest. The Sparse NMF MCC and its modified version show sharp and consistent frequency localization. The Classic NMF shows more variation, and β-HALS NMF produces a flatter distribution with lower amplitude (the background level is slightly higher).

In the middle row (σ=1.2), the increased noise degrades the performance of all methods; however, the Sparse NMF MCC variants still preserve the dominant frequency structure across most runs. The classic NMF exhibits more scattering across frequencies. The β-HALS NMF allows for detecting the proper frequency; however, the background of the noise also increased significantly.

In the bottom row (σ=2.0), corresponding to the lowest SNR, all methods struggle to extract meaningful components due to the extremely noisy environment. In the Sparse NMF MCC method, the frequency localization is unclear or inconsistent across iterations. Similarly, both Classic NMF and β-HALS NMF result in highly scattered and noisy outputs with no dominant spectral focus. These results indicate that, although the proposed Sparse NMF MCC methods are relatively more robust, all methods experience performance degradation under very low SNR conditions.

**Figure 10:**
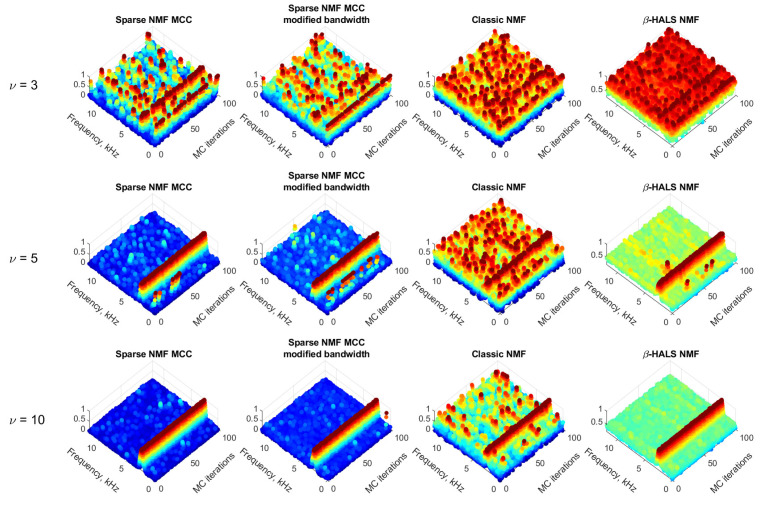
100 Simulated signals in the presence of a different range of heavy-tailed noise (Student’s t).

The Figure 10 presents the Monte Carlo analysis for 100 independent iterations under heavy-tailed noise modeled by the Student’s *t*-distribution with different degrees of freedom: ν=3,5,10. As ν increases, the tail behavior becomes lighter and the distribution approaches Gaussian. This analysis is designed to evaluate the robustness and consistency of various NMF-based filtering methods in the presence of increasingly impulsive noise.

Each row corresponds to a different value of ν, representing various levels of impulsiveness. The columns show the four filtering techniques: Sparse NMF MCC, Sparse NMF MCC with modified bandwidth, Classic NMF, and β-HALS NMF. The vertical axis shows the normalized spectral amplitude, while the horizontal axes represent frequency and Monte Carlo iterations.

In the top row (ν=3), the noise is strongly heavy-tailed (highly impulsive). All methods struggle to maintain frequency localization, and the spectral responses are scattered across iterations. Although Sparse NMF MCC and its modified variant show slightly better concentration compared to Classic NMF and β-HALS NMF, none of the methods achieve stable or reliable frequency tracking under such conditions.

In the middle row (ν=5), the impulsiveness is moderate. The Sparse NMF MCC methods begin to show more consistent localization around the frequency of interest, while Classic NMF displays scattered outputs and poor reproducibility. Although the β-HALS NMF shows a correct and consistent bandwidth, the noise level is significantly increased, which substantially degrades its performance.

In the bottom row (ν=10), the noise distribution is much closer to Gaussian. The results show more precise and consistent frequency patterns for all methods; however, the performance of Sparse NMF MCC remains the most robust, with concentrated frequency tracking across all iterations. These findings confirm that while all methods benefit from reduced impulsiveness, the proposed Sparse NMF MCC framework offers the best trade-off between robustness and consistency under heavy-tailed noise conditions.

The Monte Carlo simulations indicate that the Sparse NMF MCC method and those with the modified bandwidth, offers consistent and proper results under both Gaussian and impulsive noise conditions. It achieves better frequency localization and lower variability of the background level compared to the other tested methods.

In addition, the efficiency calculations have been carried out. Simulations were conducted for the 36 equally spaced values of the parameter ν, ranging from 3 to 10.

To determine the threshold for distinguishing whether a given signal comes from the healthy machine, healthy signals were simulated values (36 × 150 signals), and the ENVSI were computed (denoted $ENVSI_{H}$). A threshold value is defined as $1.25\times \max(ENVSI_{H})$.

**Figure 11:**
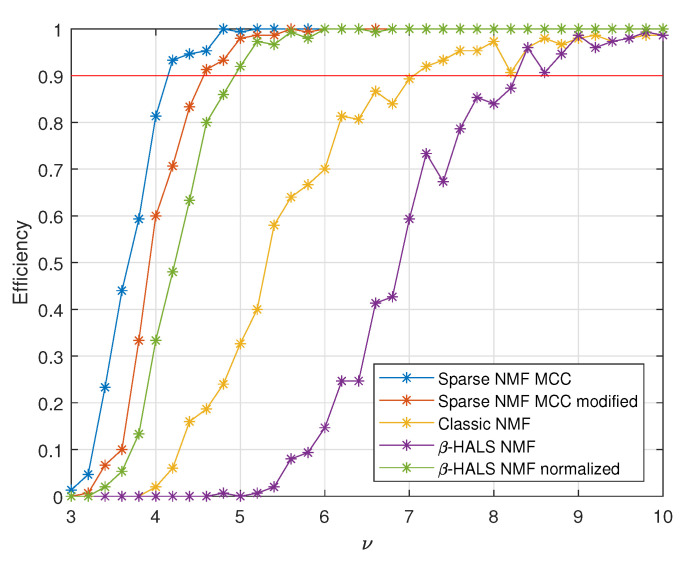
Efficiency calculation. The red line indicates an efficiency level of 90% correct results.

In Figure 11, an efficiency value of 1 indicates that the fault was correctly detected in 100% of simulations. The performance of the proposed methods, Sparse NMF MCC and Sparse NMF MCC with modified bandwidth, was compared to Classic NMF and β-HALS NMF. Although β-HALS NMF is designed for non-Gaussian signals, it exhibits a high background level, which results in low ENVSI values. To compare, the selected base feature in β-HALS NMF was normalized by subtracting its minimum value.

### 3.7. Sensitivity Analysis

**Figure 12:**
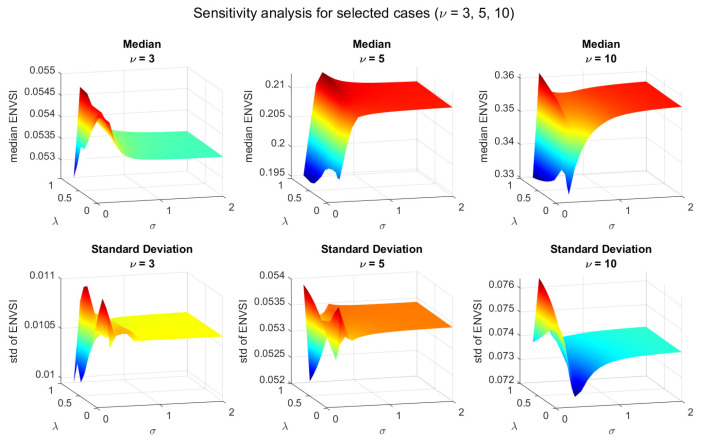
Parameter sensitivity of the NMF-based method under a different range of heavy-tailed noise (Student’s t).

The Figure 12 presents the results of the sensitivity analysis of the NMF-based algorithm with respect to its two model parameters: λ and $\sigma_{k}$. The algorithm’s performance is assessed using the ENVSI. As can be seen, 19 $\sigma_{k}$ values from the range between 0 and 2 and 14 λ parameters from the range between 0 and 1 were taken into account. As in the previous results, each column corresponds to test signals generated with a different parameter ν, namely 3, 5, and 10. The upper row of plots shows the median ENVSI values obtained from 30 Monte Carlo simulations, while the lower row illustrates the corresponding standard deviations. For ν=3, where the simulated signals exhibit heavy tails and high variability, the algorithm performs inconsistently, and ENVSI values remain low regardless of parameter choice. In general, larger values of λ lead to improved algorithm efficiency. Moreover, the $\sigma_{k}$ in the range between 0.3 and 0.7 allows for obtaining the most promising results. This demonstrates that properly tuned λ and $\sigma_{k}$ significantly enhance the robustness of the NMF-based method, especially when the input data deviates from Gaussian behavior.

## 4. Real Data Analysis

To validate the proposed method under real-world conditions, three distinct case studies are presented. These include: (1) acoustic monitoring of belt conveyor idlers in an operational industrial environment, (2) vibration analysis of a copper ore crusher subjected to heavy-duty loads, and (3) controlled fault diagnosis using a laboratory-scale test rig. Each case offers a unique combination of signal characteristics, fault types, and measurement setups, providing a comprehensive assessment of the robustness and adaptability of the proposed approach to both acoustic and vibration signals under practical constraints.

### 4.1. Case 1: Belt Conveyor Acoustic Signal

**Figure 13:**
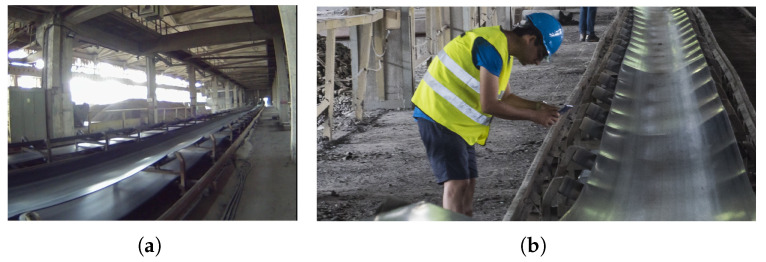
Conveyor belt and signal acquisition setup. (**a**) Conveyor belt system. (**b**) Acquiring acoustic signal from conveyor belt.

Idlers are rotating components of belt conveyors designed to support the moving belt (see Figure 13a). Each idler consists of a shaft, two bearings, and a protective coating. Given the large number of idlers in operation, a fast, contactless method to evaluate the condition of their bearings is crucial. To address this need, an experiment was conducted on a fully operational conveyor system. Due to the practical limitations, in-depth visual inspection was not possible, but the characteristics of the signal indicated the presence of localized damage.

For the experiment, audio recordings were taken from each idler using a standard smartphone (see Figure 13b). Each recording lasted approximately 10 s and had a sampling frequency of 48 kHz. The sound was extracted from video recordings using MATLAB’s built-in function. The captured signals exhibited characteristics indicative of faults, making them suitable for further analysis.

### 4.2. Case 2: Copper Ore Crusher

**Figure 14:**
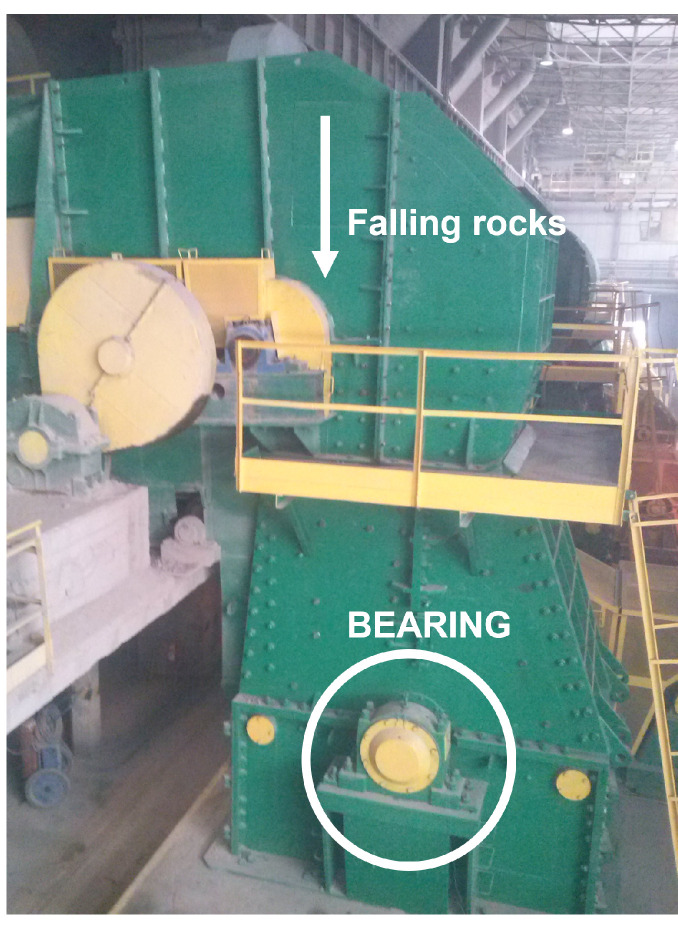
Copper ore crusher machine.

An ore crusher used in the mining industry (see Figure 14) serves as a case study to demonstrate the advantages of the proposed approach. The crusher operates under variable load and speed conditions due to time-varying load fluctuations imposed by the unknown volume and size of the material pieces that impose resistance on the machine. A vibration signal was measured on the casing of the rolling element bearing that supports the crusher’s main shaft. The signal was recorded using Endevco accelerometers, model 751-10, (New York, United States) and a data acquisition system managed through LabVIEW Signal Express 2013 software. Vibration signals were collected during normal operation, and an example was selected in which the crusher was fully loaded, including oversized pieces of material. The parameters of the signal are as follows: a sampling frequency of 25 kHz and a total duration of approximately 2 min, although only a 10-s segment is analyzed in this paper.

In the industry, it is challenging to obtain representative signals from the machine with a faulty bearing. The special case of the crusher underscores a critical engineering issue: its impulsive loading characteristics mean that even minimal damage can quickly escalate, potentially causing catastrophic system failure. To ensure a controlled experiment, data from a healthy bearing was used as a baseline, while a cyclic impulsive signal (representing the SOI) was added to simulate a local fault. This approach allows for testing various fault severities, including scenarios where the SOI is either fully obscured by background noise or just barely visible in the raw signal but remains difficult to detect. The fault frequencies for the bearing (SKF 23264) are listed in Table 2, and the analysis focuses on an inner race fault within one of the bearings.

### 4.3. Case 3: Test Rig

**Figure 15:**
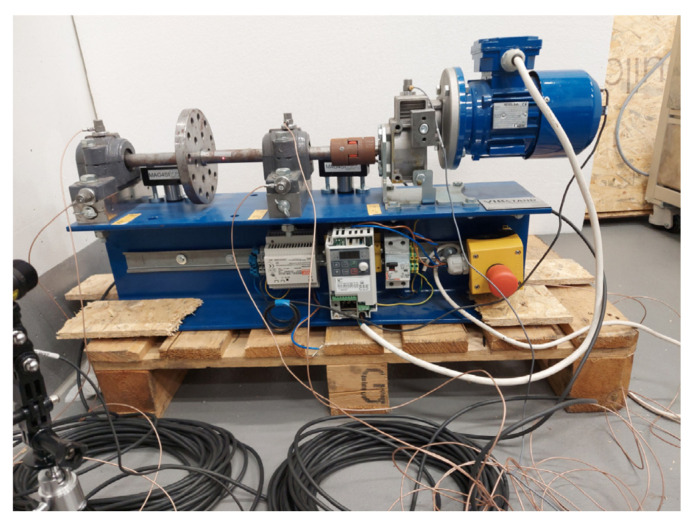
Test rig used in the experiment.

The experimental data were acquired using a dedicated test rig consisting of an electric motor, gearbox, couplings, and two bearings, as shown in Figure 15. To simulate realistic fault conditions, one of the bearings was intentionally damaged. Acoustic signals were recorded at a sampling rate of 50 kHz using a Brüel & Kjær 4189 microphone (Nærum, Denmark) along with a Kistler LabAmp 5165 (Sindelfingen, Germany) A data acquisition system. Throughout the experiment, the rotational speed was maintained at a constant 1041 rpm. The bearings used were SKF 1205 EKTN9 models, with one exhibiting a localized defect on the outer race. The characteristic frequencies associated with the bearing components are summarized in Table 3, where the outer race defect frequency is highlighted at 91.11 Hz.

### 4.4. Results for Real Dataset

**Figure 16:**
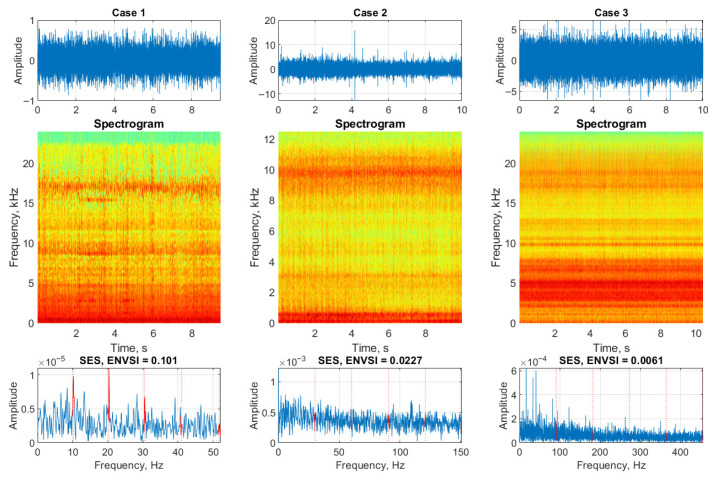
The time series, spectrogram, and SES for Case 1, Case 2, and Case 3, respectively.

In the following section, preliminary analyses using well-established approaches are presented for the three real-world case studies shown in Figure 16. The Figure 16 illustrates the baseline signal analysis for the three real-world case studies introduced earlier. Each column corresponds to one case: Case 1 (belt conveyor), Case 2 (copper ore crusher), and Case 3 (test rig with seeded fault). The figure is structured into three rows to present a comparative overview of the time-domain signals, spectrograms, SES.

In the first row, the raw acoustic or vibration signals are shown for each case. Across all three cases, the impulsive component is not clearly distinguishable in the time domain due to the influence of background noise and operating variability. In particular, Case 2 and Case 3 exhibit relatively stronger amplitude fluctuations, likely resulting from load or structural resonance effects.

The second row presents the spectrograms of the signals, computed using MATLAB’s spectrogram function with parameters appropriate for each sampling rate. While energy concentration across frequency bands can be observed, there is no visible evidence of repetitive impulsive signatures or modulation bands in any of the cases. This highlights the difficulty of detecting bearing-related faults in raw measurements without pre-processing or enhancement, especially under operational noise.

The third row shows the SES along with the calculated ENVSI values. For Case 1, several periodic peaks are weakly visible and the ENVSI reaches a moderate value of 0.101, indicating a low level of modulation sparsity. In Case 2, the SES is almost flat and noisy, with an ENVSI of just 0.0227, making fault-related frequency components indistinct. Case 3 shows even lower sparsity (ENVSI = 0.0061), and no clear periodic components are observed. These results confirm that raw signal energy analysis and conventional envelope techniques alone are insufficient for reliable fault diagnosis in complex industrial and experimental environments.

**Figure 17:**
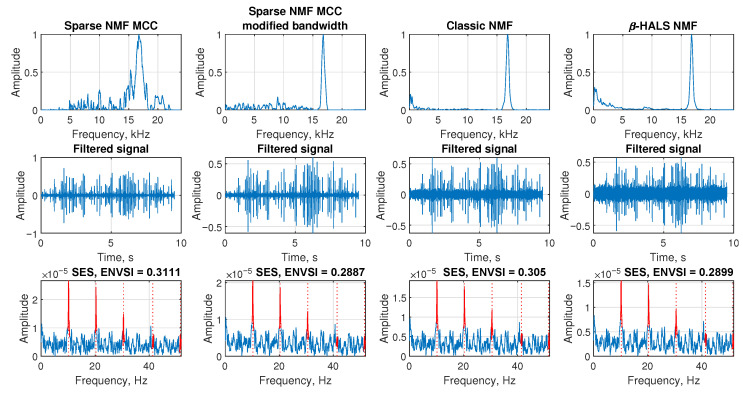
Filtering results for Case 1. Comparison of NMF-based methods using frequency response, filtered signal, and SES with ENVSI values.

**Figure 18:**
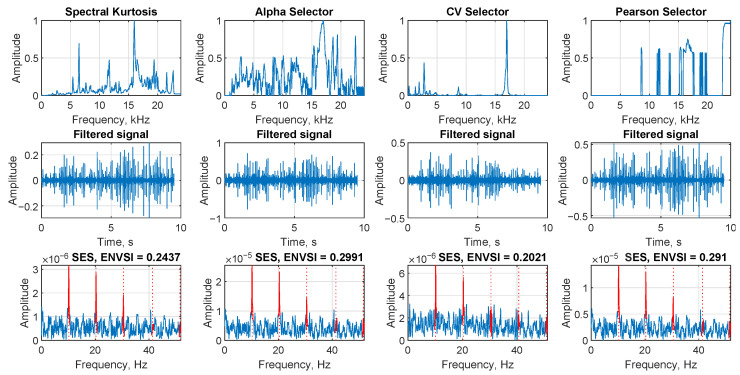
Filtering results for Case 1. Comparison of four selectors, filtered signal, and SES with ENVSI values.

**Figure 19:**
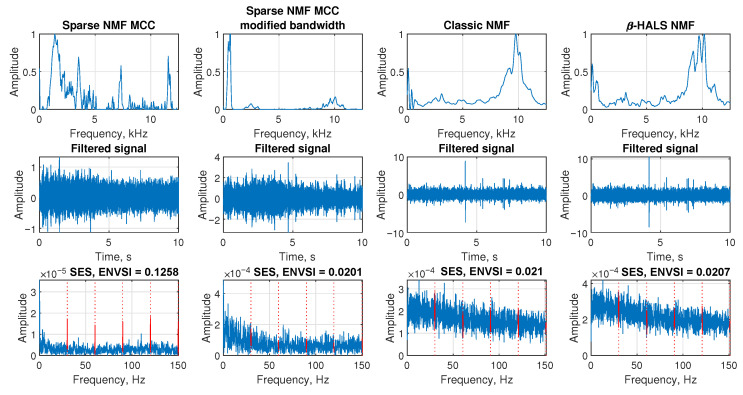
Filtering results for Case 2. Comparison of NMF-based methods using frequency response, filtered signal, and SES with ENVSI values.

**Figure 20:**
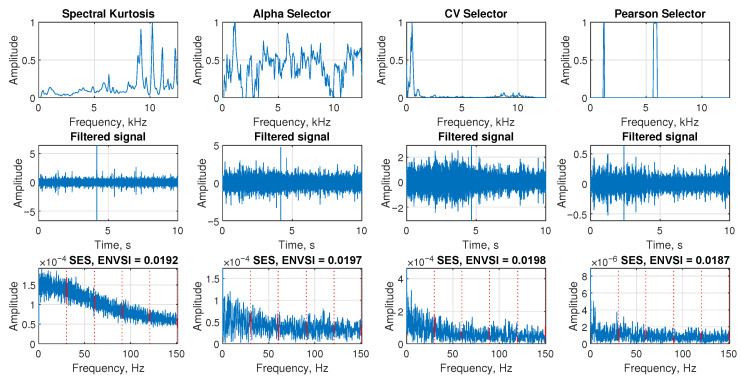
Filtering results for Case 2. Comparison of four selectors, filtered signal, and SES with ENVSI values.

**Figure 21:**
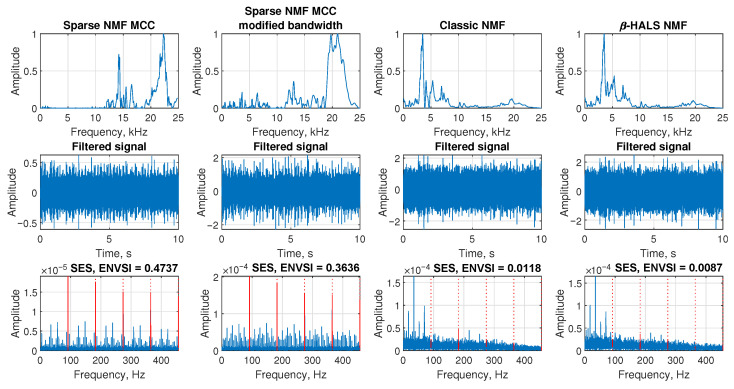
Filtering results for Case 3. Comparison of NMF-based methods using frequency response, filtered signal, and SES with ENVSI values.

**Figure 22:**
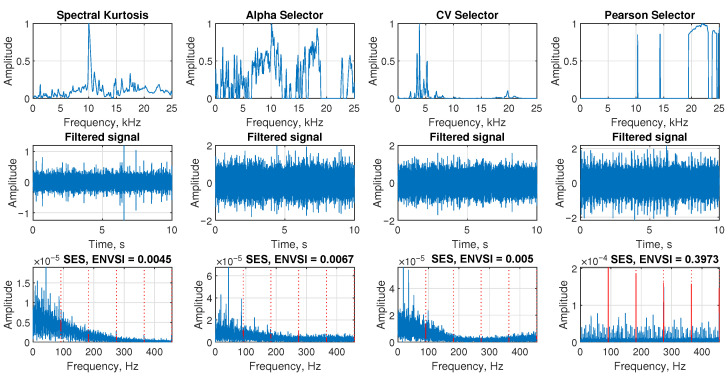
Filtering results for Case 3. Comparison of four selectors, filtered signal, and SES with ENVSI values.

The Figure 17, Figure 19, and Figure 21 present the performance of four NMF-based filtering methods applied to the signals from Case 1, Case 2, and Case 3, respectively. Each column corresponds to one method: Sparse NMF MCC, Sparse NMF MCC with modified bandwidth, Classic NMF, and β-HALS NMF. Similarly, the Figure 18, Figure 20, and Figure 22 present the performance of four selectors selected for comparison applied to the signals from Case 1, Case 2, and Case 3, respectively. Each column corresponds to one selector: spectral kurtosis, alpha selector, CV selector, and Pearson selector. The figures are organized into three rows that visualize the selected informative frequency band, the filtered time-domain signal, and the resulting SES with the ENVSI.

The Figure 17 presents the performance of four NMF-based filtering methods applied to the acoustic signal from Case 1 (belt conveyor idler). In the first row, the extracted frequency component is shown. All methods identify a band around 15–18 kHz. The modified bandwidth version presents the narrowband selectivity. In contrast, Classic NMF and β-HALS NMF exhibit sharper peaks but with higher values in low-frequency bands, which results in higher noise levels in the filtered signals. The Sparse NMF MCC has lower selectivity in the 15–18 kHz range; however, it obtains values around zero in the low-frequency band. It gives a lower noise level in the filtered signal and a higher ENVSI value.

The second row shows the corresponding time-domain filtered signals. All methods recover an amplitude-modulated impulsive structure, though with varying levels of noise suppression. Sparse NMF MCC results in a cleaner impulsive pattern, while the other three methods produce noisier outputs with lower peak-to-background contrast.

The third row displays the SES for each filtered signal, along with the corresponding ENVSI value. All four methods successfully reveal a periodic pattern in the frequency domain. Sparse NMF MCC achieves the highest ENVSI (0.3111), followed by Classic NMF (0.305), β-HALS NMF (0.2899), and Sparse NMF MCC (mod. BW) (0.2887). Although the ENVSI values are relatively close, the SES generated by Sparse NMF MCC provides clearer periodic peaks. These results demonstrate that all methods are capable of detecting the impulsive modulation associated with the fault, but the proposed Sparse NMF MCC offers a slight edge in terms of clarity and sparsity.

The Figure 18 presents the results of four selectors calculated for the signal for Case 1. In the first row, the shapes of the obtained selectors are presented. The second row shows the resulting time-domain signals after filtering. The third row presents the SES of the filtered signals along with the corresponding ENVSI values. The alpha selector and Pearson selector present the highest ENVSI value equal to 0.2991 and 0.291, respectively. Note that it is still a lower value than the ENVSI value calculated for the signal filtered by the proposed Sparse NMF MCC.

The Figure 19 presents the results of applying four NMF-based filtering methods to the vibration signal collected from the copper ore crusher (Case 2). In the first row, the filter characteristics of the extracted components are displayed. Classic NMF and β-HALS NMF identify the informative band as the one in which non-cyclic impulses occur. In the case of Sparse NMF MCC with modified bandwidth, the most informative band is indicated as being below 1 kHz. Finally, the Sparse NMF MCC indicates the band in which cyclic impulses associated with damage occur. Despite the appearance of values in higher bands, it is the only one that correctly shows the information band.

The second row shows the time-domain signals after filtering. The impulsive content remains buried in noise, especially in Classic NMF and β-HALS NMF, where the filtered signals exhibit large amplitude spikes that do not follow a periodic pattern. Sparse NMF MCC method suppresses background variation more effectively, though the recovered signal is still far from clean, it allows damage to be detected.

In the third row, the SES plots for each method are shown. Sparse NMF MCC achieves the highest ENVSI value (0.1258), with faint periodic peaks corresponding to fault-related frequencies. The other three methods produce noisy SES plots with little distinguishable structure and low ENVSI values: 0.0201 for Sparse NMF MCC (mod. BW), 0.021 for Classic NMF, and 0.0207 for β-HALS NMF. These results demonstrate that while the environment is too noisy for reliable extraction using conventional methods, the proposed Sparse NMF MCC still provides the most promising outcome in terms of modulation sparsity and fault feature enhancement.

The Figure 20 presents the results of four selectors calculated for the signal for Case 2. As can be observed in the first row, none of the presented selectors cannot select the proper informative frequency band. Both spectral kurtosis and methods dedicated to impulsive noise data were unable to indicate the IFB correctly. As a result, both the filtered signals and the SES failed to represent the fault component effectively, and the ENVSI values obtained are very low.

The Figure 21 presents the filtering results for Case 3, which involves acoustic measurements from a test rig containing a bearing with an outer race defect. In the first row, the spectral responses extracted by each method are shown. Both Sparse NMF MCC methods capture a narrow and focused band around 20–23 kHz, corresponding to the fault-induced modulation. In contrast, Classic NMF and β-HALS NMF identify much broader and noisier components, resulting in ineffective separation of the impulsive content.

In the second row, the filtered signals in the time domain are visualized. Sparse NMF MCC produces a clean signal with clearly distinguishable impulsive events. The modified bandwidth version also reveals regular impulsive patterns but includes slightly higher background noise. Classic NMF and β-HALS NMF produce filtered signals that are more contaminated with noise, lacking any distinct repetitive features.

The third row shows the SES and ENVSI values. Sparse NMF MCC yields the highest ENVSI (0.4737), followed by its modified bandwidth version (0.3636), both showing sharp peaks at the fault frequency and its harmonics. Classic NMF and β-HALS NMF achieve significantly lower ENVSI values (0.0118 and 0.0087, respectively), and the periodic peaks are barely visible. These results confirm that Sparse NMF MCC is the most effective method for isolating fault-related modulations in controlled laboratory conditions.

The Figure 22 presents the results of four selectors calculated for the signal for Case 3. Spectral kurtosis, alpha selector, and CV selector do not allow the correct IFB to be obtained. They focus on other frequency bands. The Pearson selector, in contrast, accurately indicates the proper bandwidth and is highly selective. This is evident in both the filtered signal, where cyclic impulses are visible, and on the SES. The ENVSI value obtained in this case is 0.3973.

**Figure 23:**
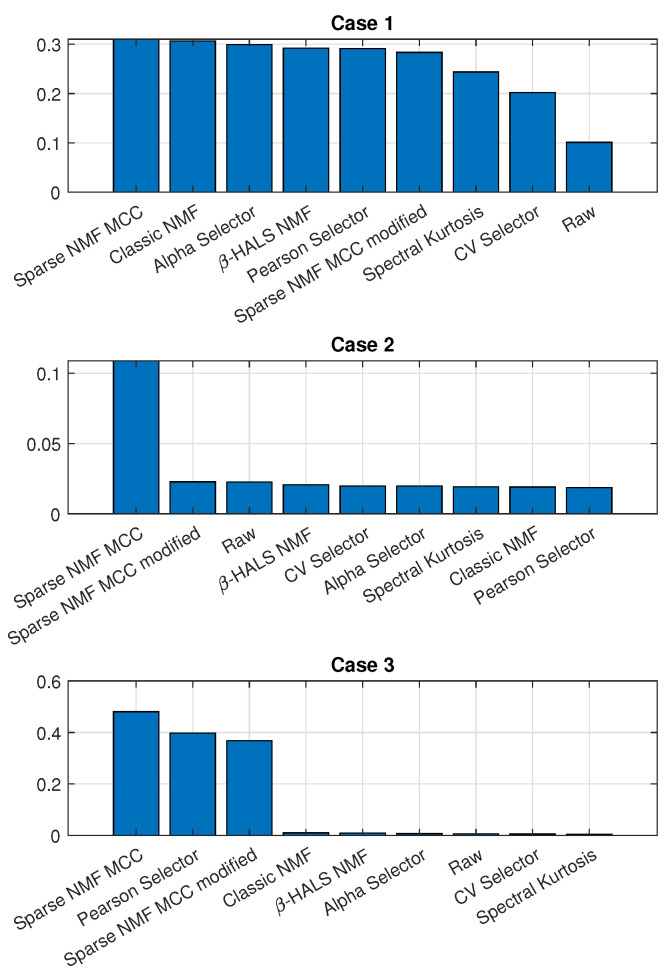
Comparison of the obtained ENVSI values for all analyzed methods for signals corresponding to the Case 1, Case 2, and Case 3, respectively.

Figure 23 presents a comparative analysis of the ENVSI values obtained for all evaluated methods across three different cases. The ENVSI serves as an efficiency indicator, quantifying how well each approach enhances the visibility of fault-related components in the squared envelope spectrum.

In Case 1, all methods yield relatively similar ENVSI values, enabling the detection of damage; however, the Sparse NMF MCC and Classic NMF slightly outperform the others. In Case 2, the Sparse NMF MCC has the highest value of the ENVSI, and it is the only one that allows for the detection of damage. In Case 3, both Sparse NMF MCC and its modified version obtain a high value of ENVSI. In this case, the Pearson selector also enables the detection of damage. The results from the analysis of real data show that the proposed Sparse NMF MCC consistently achieves better diagnostic performance in all cases, confirming its robustness and efficiency in extracting fault-related information from complex vibration signals.

Moreover, to ensure fair comparison, all NMF-based methods were tested both with their default initialization schemes (as provided in their standard implementations) and with NNDSVD initialization.

The comparative results for Cases 1, 2, and 3 are summarized in Table 4. The comparison has been made with the MATLAB implementation of the classical NMF (default) and with the random initialization in β-HALS NMF. The use of NNDSVD initialization did not significantly affect the quality of the obtained results for Classical NMF and β-HALS (the ENVSI parameter values changed by at most 0.01). However, this significantly affects the results obtained with sparse MCC NMF for Case 2, where NNDSVD initialization enables damage detection. The performance of the contribution of each component of the proposed algorithm is presented in Appendix A.

The main hyperparameters and their empirically selected ranges for the three experimental case studies are summarized in Table 5. The NMF rank r was chosen based on the number of components to include in the signal: one class for damage, one for noise, and two for non-Gaussian noise. The sparsity coefficient and the MCC kernel bandwidth were tuned by a trial-and-error process guided by the ENVSI and visual inspection of the reconstructed components, also based on the rule in Section 2.6.

### 4.5. Discussion, Limitations, and Future Work

The proposed Sparse NMF-MCC demonstrates strong robustness under both Gaussian and heavy-tailed noise conditions and achieves effective separation of fault-related components in both simulated and real-world datasets. However, as with any decomposition-based method, certain limitations and potential failure cases should be acknowledged. The performance may degrade when impulsive fault signatures overlap strongly with broadband background noise, when the decomposition rank is not appropriately chosen, or when fault impulses are extremely weak relative to dominant structural resonances. These conditions can lead to partial mode mixing or reduced separability among NMF components.

Moreover, the current method requires manual selection of hyperparameters such as the MCC bandwidth, sparsity coefficient, and decomposition rank, which can influence performance across different datasets. In future work, these parameters can be optimized automatically using optimization or learning-based algorithms to adapt the model to varying signal and noise characteristics. The present implementation also relies on the STFT representation; extending it to adaptive time–frequency or tensor-based frameworks could further enhance its capability to capture complex non-stationary patterns.

While the current study intentionally focuses on the NMF family to analyze and improve its robustness under heavy-tailed and non-cyclic impulsive noise, deep-learning frameworks represent an exciting complementary direction. In our future research, we plan to integrate the proposed Sparse NMF-MCC within hybrid or deep-learning-based architectures to combine the interpretability and noise robustness of model-based decomposition with the adaptability of data-driven learning. Finally, real-time and online implementations represent promising directions for practical industrial monitoring and fault-diagnosis systems.

## 5. Conclusions

In this paper, we propose a robust sparse NMF method based on the maximum correntropy criterion for the automatic identification of signals of interest in bearing fault detection. The proposed approach addresses key limitations of traditional NMF-based decomposition techniques by introducing robustness against outliers and heavy-tailed noise via the correntropy measure. In addition, the inclusion of an $\ell_{1}$-norm sparsity constraint promotes the isolation of impulsive features typically associated with mechanical faults.

Comprehensive Monte Carlo simulations under various noise conditions, including both Gaussian and heavy-tailed distributions, demonstrated the superior ability of the proposed method to extract cyclic impulsive components embedded in noisy environments. These results were further validated through real-world case studies involving conveyor belt systems, a copper ore crusher, and data from a controlled test rig. In all cases, the method successfully identified and enhanced fault-related frequency bands, even under challenging noise scenarios.

Overall, the proposed Sparse NMF MCC framework represents a significant advancement in automated fault detection for rotating machinery. Its ability to robustly extract informative components in the presence of noise makes it particularly well-suited for industrial applications where conventional techniques often fail.

## Figures and Tables

**Table 1:** Parameters used for the spectrogram generation.

Parameter	Value
Window	hamming(256)
Overlap	180
FFT Points	512
Sampling Frequency (Fs)	25,000

**Table 2:** Characteristic frequencies of 23264 CCK/W33 bearing.

Description	Value
Shaft speed frequency	3 Hz
Cage defect frequency (FTF)	1.3 Hz
Ball spin frequency (BSF)	10.6 Hz
Inner race defect frequency (BPFI)	30.7 Hz
Outer race defect frequency (BPFO)	23.3 Hz
Rolling element defect frequency	21.1 Hz

**Table 3:** Characteristic frequencies of 1205 EKTN9 bearing.

Description	Value
Shaft speed frequency	17.35 Hz
Cage defect frequency (FTF)	7 Hz
Ball spin frequency (BSF)	42.75 Hz
Inner race defect frequency (BPFI)	134.44 Hz
Outer race defect frequency (BPFO)	91.11 Hz
Rolling element defect frequency	85.5 Hz

**Table 4:** Comparison of NMF with different initializations and selector results for Cases 1–3, respectively. The highest value in each column has been bolded.

Method	Type	Case 1	Case 2	Case 3
NMF Sparse MCC	Random	0.2803	0.0196	0.4650
	NNDSVD	**0.3111**	**0.1258**	**0.4737**
Sparse NMF MCC Modified Bandwidth	Random	0.3011	0.0228	0.3188
	NNDSVD	0.2887	0.0201	0.3636
Classic NMF	Default	0.3051	0.0197	0.0136
	NNDSVD	0.3050	0.0210	0.0118
β-HALS NMF	Random	0.2927	0.0208	0.0088
	NNDSVD	0.2899	0.0207	0.0087
Spectral Kurtosis	–	0.2437	0.0192	0.0045
Alpha Selector	–	0.2991	0.0197	0.0067
CV Selector	–	0.2021	0.0198	0.0050
Pearson Selector	–	0.2910	0.0187	0.3973

**Table 5:** Key hyperparameters and empirical selection ranges used in the Sparse NMF-MCC algorithm for the three experimental case studies.

Parameter	Symbol	Case 1	Case 2	Case 3
		(Conveyor)	(Ore Crusher)	(Bearing Rig)
Sparsity coefficient	λ	1	1	1
MCC kernel bandwidth	$\sigma_{k}$	0.3	0.3	0.3
Factorization rank	*r*	4	4	4

**Figure A1:**
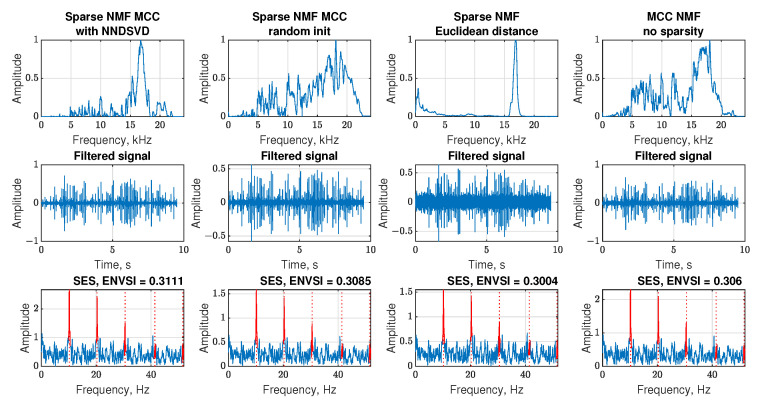
Filtering results for Case 1.

**Figure A2:**
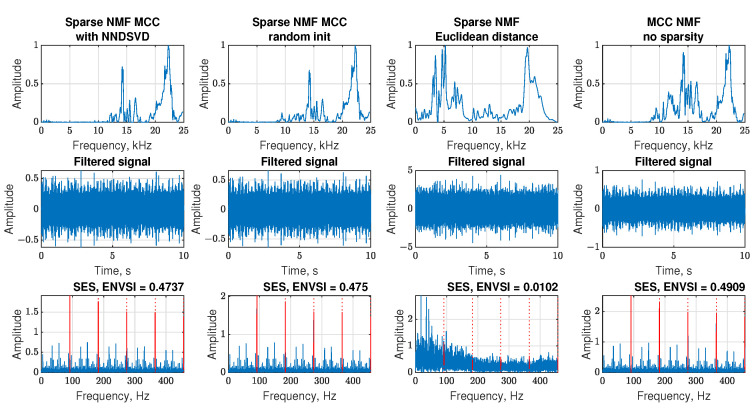
Filtering results for Case 3.

**Figure A3:**
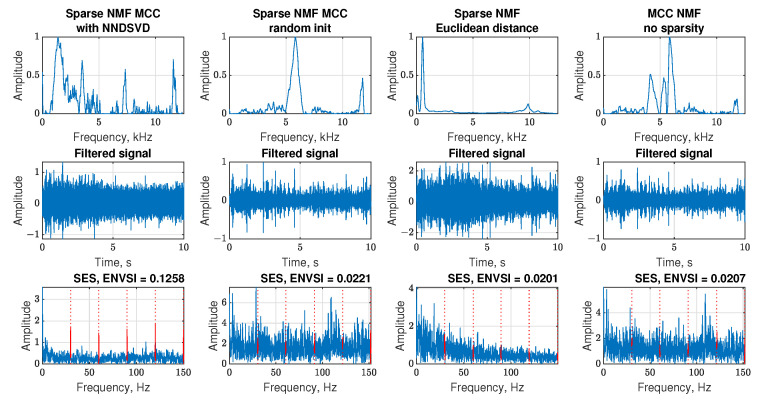
Filtering results for Case 2.

## Data Availability

The measurement data presented in this study are not publicly available due to restrictions of privacy.

## Appendix A

The performance of the contribution of each component of the proposed algorithm are presented. The results are presented in four columns, each corresponding to the impact of a different component of the algorithm. The first column shows the results for Sparse NMF MCC with NNDSVD initialization, the second for Sparse NMF MCC with random initialization, the third for Sparse NMF with Euclidean distance, and the fourth for MCC NMF without sparsity.

In Figure A1, all methods yield the correct result, and the differences in ENVSI values are not significant; however, the proposed methods produce the highest ENVSI value.

In Figure A2, the Sparse NMF with the Euclidean distance does not allow for obtaining the correct result. It suggests that the use of MCC is reasonable.

Finally, as shown in Figure A3, only the proposed procedure employing Sparse MCC NMF with NNDSVD initialization yields the correct result. The other methods in this comparison fail to produce filter characteristics conducive to obtaining a valid outcome. In summary, each element of the approach is important.

## Appendix B

The list of key variables and parameters used in the proposed methodology has been presented in Table A1.

**Table A1:** Variables and Parameters Used in the Proposed Methodology.

Symbol	Description
y	One-dimensional discrete data (raw input signal)
$STFT_{t,f}$	Short-Time Fourier Transform at time *t* and frequency *f*
*w*	Window function
*L*	Number of samples in the analyzed segment (window length)
*N*	Number of points used for Discrete Fourier Transform (DFT)
*j*	Imaginary unit
$F_{s}$	Sampling Frequency
$S\in R_{+}^{m\times n}$	Spectrogram (absolute value of STFT), the non-negative input matrix,
m,n	Dimensions (rows, columns) of the input matrix *S*
*r*	Rank of factorization
λ	Sparsity regularization parameter
maxIter	Maximum number of iterations in the Sparse NMF MCC algorithm
$W\in R_{+}^{m\times r}$	Basis matrix
$H\in R_{+}^{r\times n}$	Coefficient matrix
E	Error matrix
*V*	Correntropy
$\kappa_{\sigma_{k}}\left(\cdot \right)$	Gaussian kernel function in Maximum Correntropy Criterion (MCC)
$\sigma_{k}$	Kernel bandwidth parameter ($\sigma_{k}>0$) in MCC
${∥\cdot ∥}_{1}$	$l_{1}$ norm
Ω	MCC weight matrix
⊙	Hadamard product (element-wise multiplication)
⊘	Hadamard division (element-wise division)
ϵ	Small constant introduced to prevent division by zero
ENSVI	Envelope Spectrum Indicator
SES	Squared Envelope Spectrum (SES)
s	Signal of Interest (SOI) component
n	Noise component
ν	Degrees of freedom (for Student’s t-distribution, modeling heavy-tailed noise)
*A*	Magnitude/Amplitude of the sinusoidal component in SOI
$f_{c}$	Frequency of the sinusoidal component in SOI
$\alpha_{s}$	Damping factor
δ(·)	Dirac delta function
$T_{p}$	Nominal period of the signal
$\Delta T_{p}\left(t\right)$	Slow variations in the period due to jitter effects
$\phi_{s}$	Initial phase shift
*	Convolution operation
σ	standard deviation in Gaussian noise
